# Anti-Inflammatory Effects of *Gynura procumbens* on RAW264.7 Cells via Regulation of the PI3K/Akt and MAPK Signaling Pathways

**DOI:** 10.1155/2022/5925626

**Published:** 2022-04-16

**Authors:** Ming-Yuan Cao, Jing Wu, Lei Wu, Zhen Gu, Ju-Wu Hu, Chuan-Qi Xie, Wei Xiong

**Affiliations:** ^1^Institute of Applied Chemistry, Jiangxi Academy of Sciences, Nanchang 330096, China; ^2^School of Food Science and Engineering, Jiangxi Agricultural University, Nanchang 330000, China

## Abstract

*Gynura procumbens* is a traditional herb and food extensively cultivated in China and Southeast Asian countries. In this work, the crude extract (CE) of *G. procumbens* was purified with macroporous resin to obtain the refined fraction, and its anti-inflammatory activity was compared with that of CE. Moreover, the detailed mechanisms of anti-inflammatory activity were also investigated for the first time. The results indicated that CE was more effective in anti-inflammatory activity and it could reduce the secretion of NO, TNF-*α*, and PGE2 via decreasing the iNOS, TNF-*α*, and COX-2 genes transcription and related proteins translation, which were associated with the inhibition of AP-1 and NF-*κ*B nuclear translocation and downregulation of PI3K/Akt and MAPK signaling pathways. In conclusion, the extract of *G. procumbens* has a promising potential in inflammation-related disorders alleviation, and these findings could provide the basis for the comprehensive utilization of *G. procumbens* and the new functional food development.

## 1. Introduction

Inflammation is a normal physiological response that our bodies have against the external stimuli, relating to tissue damage and diseases [1], which is also regarded as a “silent health killer” and broadly divided into acute inflammation and chronic inflammation. Acute inflammation is self-resolving by nature, while chronic inflammation often occurs for a long time and does not resolve easily inducing arthritis, cardiovascular diseases, diabetes, Alzheimer's disease, cancer, and other chronic diseases [2, 3], in a sense, inflammation. Macrophages, a type of immune cells, are an important barrier against most diseases in our body. It could increase the nitric oxide (NO) release and the secretion of inflammatory factors consisting of prostaglandin E2 (PGE2), interleukin-1*β* (IL-1*β*), and tumor necrosis factor-*α* (TNF-*α*) in order to initiate the response of inflammation when stimulated by tissue injury and external microorganism infection [4]. These release of proinflammatory factors and the expressions of related genes are inextricably linked to intracellular signaling. Phosphoinositide 3-kinase (PI3K)/Akt and mitogen-activated protein kinase (MAPK) are crucial signaling pathways in inflammatory response; once activated, it will cause a cascade of downstream proteins. The transcription factors comprising nuclear factor-*κ*B (NF-*κ*B) and activator protein 1 (AP-1) are pivotal components in downstream signaling, functioning at the merging end point of diverse signaling pathways [5, 6]. Hence, any of the components or substances which can downregulate these signaling pathways and inhibit the release of inflammatory cytokines are greatly beneficial to the treatment of inflammation. Nonsteroidal anti-inflammatory drugs have been used commonly in the clinical treatment for pains and inflammations, whereas these drugs have some side effects including nausea, gastric bleeding, vomiting, and liver damage for long term use [2, 7]. Thus, the exploitation of effective natural anti-inflammatory drugs is the focus of current research.

*Gynura procumbens* (Lour.) Merr. is a traditional herb and food extensively cultivated in China and Southeast Asian countries, belonging to the Asteraceae family [8, 9]. Particularly, it is extensively referred to as “Sambung Nyawa” in Malaysia, meaning longevity and “cure-all” [10]. The chemical constituents of *G. procumbens* include steroids, caffeoylquinic acids, fatty acids, flavonoids, and terpenoids, which contribute for its bioactivities comprising anti-inflammatory, antioxidative, antithrombotic, and analgesic [11–13]. Thus, *G. procumbens* have been broadly used in modern medicine to treat many diseases, such as diabetes, cancer, arthritis, gout, and cardiovascular disease [14–16]. In addition, the leaves of *G. procumbens* (GPL) are proven to be nontoxic and are used as a material to make toothpaste, skin cream, candy, and tea. The National Health Commission of the PRC also approved *G. procumbens* as a new food resource in 2012 [17, 18]. Taken together, GPL shows promising potential from the perspective of whether it has edible or medicinal value.

To our knowledge, the anti-inflammatory activity of GPL has been reported in several research studies, but its detailed mechanisms are poorly involved. Although the anti-inflammatory activities of single compounds in GPL including chlorogenic acid and astragalin have been reported, specific active compounds cannot fully represent the crude extract. Moreover, the whole leaves of *G. procumbens* are often consumed directly as fresh vegetables in folk, so the bioactivities of the whole leaves deserve more attention. Therefore, the anti-inflammatory activity of GPL in lipopolysaccharide (LPS)-induced RAW 264.7 macrophages was studied, and the related molecular mechanisms were elucidated in this work to refine the anti-inflammation theory.

## 2. Materials and Methods

### 2.1. Materials

Fetal bovine serum (FBS) was obtained from Natocor (Cordoba, Argentina). High glucose Dulbecco's modified Eagle's medium (DMEM) was purchased from HyClone (Logan, Utah, USA). Penicillin, streptomycin, 3-(4,5-dimethylthiazol-2-yl)-2,5-diphenyltetrazolium bromide (MTT), phosphate buffer saline (PBS), LPS, and sodium dodecyl sulfate (SDS) were obtained from Sangon Biotech (Shanghai, China). Enhanced BCA Protein Assay Kit and 4,6-diamidino-2phenylindole (DAPI) were obtained from Beyotime (Shanghai, China). Griess B (1%(w/v) sulphanilamide containing 5% (w/v) H_3_PO_4_, Griess A (0.1% (w/v) N-(1-naphthyl)-ethylenediamine dihydrochloride), RIPA buffer, dimethyl sulfoxide (DMSO), and sodium nitrite were provided by Solarbio (Beijing, China). ELASA kits of TNF-*α* and PGE2 were obtained from Boster (Wuhan, China). The primary antibodies for iNOS, COX-2, p38, p-ERK, p-p38, ERK, JNK, p-JNK, *β*-actin, NF-*κ*B p65, c-Fos, p-Akt, c-Jun, PDK1, GAPDH, p-PDK1, p-I*κ*B*α*, Akt, and I*κ*B*α*, and the secondary antibody goat antirabbit (IgG) were provided by AB Clonal Biotech (Wuhan, China). Other standards and analytical grade reagents were obtained from Tianjin ZhiYuan Reagent Co., Ltd. (Tianjin, China).

### 2.2. Extraction and Preparation of Sample

The GPL were collected from Ganzhou city of China, in June 2020, and were authenticated by Prof. X.H. Li, Jiangxi Academy of Science. A voucher specimen (GPL2020-06) was stored in our laboratory. The GPL samples were dried, pulverized, and sifted, followed by two extractions (25 min each) with 65% ethanol under ultrasonication (30°C, 500 W). Next, it was concentrated under a vacuum after filtration. The lyophilization was further conducted to get crude extract (CE). Then, CE was loaded on a macroporous resin 306 and eluted with 0, 10%, 30%, 50%, 70%, and 100% aqueous ethanol for purification. The desired compounds generally elute in 30% aqueous ethanol, and this fraction was collected and marked as 30% fraction (Fr. 30%).

### 2.3. Cell Culture

RAW 264.7 cells were provided by the Key Laboratory of Pu-er Tea Science and cultured in high glucose DMEM containing 1% penicillin-streptomycin and 10% FBS. The Esco CO_2_ incubator (Shanghai, China) was used to culture the cells with 5% CO_2_ atmosphere at the constant temperature of 37°C. The cells were subcultured based on the growth.

### 2.4. Cell Viability

Cytotoxicity of CE and Fr. 30% on RAW264.7 macrophages was measured according to the MTT assay [19]. Cells (5 × 10^5^ cells/well) were seeded in 96-well plates for 24 h incubation. Next, the medium was changed and different concentrations of samples (0–100 *μ*g/mL) were added in it (except for the control group) for 24 h treatment. Next, the culture solution in every well was entirely aspirated, and 100 *μ*L serum-free medium containing 1 mg/mL MTT was further added in it for 4 h incubation. 100 *μ*L MTT stop buffer (10% SDS with 0.01 M HCl) was added and treated for 16–20 h to stop the reaction. Finally, the microplate reader (Tecan Infinite 200 Pro, Austria) was used for reading the absorbance of each well at 570 nm.

### 2.5. Determination of NO Content

The production of NO was determined by the Griess assay [19]. RAW264.7 cells (5 × 10^5^ cells/well) were cultured in a 96-well plate with 24 h incubation, and different concentrations of CE or Fr. 30% (0–100 *μ*g/mL) were added in each well (except for the control group) for 2 h pretreatment, respectively. Then, 1 *μ*g/mL LPS was added in these wells for an extra 24 h incubation, and 100 *μ*L supernatants of every well were mixed with an equal volume of Griess reagent (Griess A: Griess B, 1 : 1, v/v). After 10 min shock and reaction, the absorbance was immediately determined at 540 nm. The release of NO was calculated against the NaNO_2_ standard curve.

### 2.6. ELISA Assay

The supernatants in 6-well plate after the 2 h pretreatment with different concentrations of CE and the 24 h subsequent treatment of 1 *μ*g/mL LPS were collected for the analysis of TNF-*α* and PGE2. The contents of PGE2 and TNF-*α* were determined by ELISA kits (Boster, Wuhan, China).

### 2.7. Scanning Electron Microscopy (SEM)

RAW264.7 macrophages were cultured onto coverslips in 6-well plates (1 × 10^6^ cells/well) with 24 h incubation. The medium solution was changed, CE (50 *μ*g/mL) was added in the wells of the treatment group for 2 h of pretreatment, and 1 *μ*g/mL LPS was then added in the wells (except for the control group) for extra 24 h treatment. The cells were fixed for 1 h with 2.5% glutaraldehyde (pH 7.2–7.4) at 4°C and washed with PBS (0.1 M) for three times (5 min for each). The 1% osmium tetroxide was used to stain the cells for 1 h at 4°C, and the PBS (0.1 M) was used again for three times washing (5 min for each). Next, ethanol in 30%, 50%, 70%, 90%, and 100% gradient was used to dehydrate for two times (5 min for each). The cells were dried at the critical point and evaporated with gold [20]. Ultimately, the specific morphology of RAW264.7 macrophages was observed and assessed using the Su1510 SEM (Hitachi, Tokyo, Japan).

### 2.8. The Analysis of mRNA Expression by Semiquantitative RT-PCR and Quantitative RT-PCR

RAW264.7 macrophages (1 × 10^6^ cells/well) were cultured in 6-well plates with 24 h incubation, CE with various concentrations (0–100 *μ*g/mL) were added in each well (except control group) for 2 h of pretreatment, and 1 *μ*g/mL LPS was subsequently added for 6 h incubation. Then, total RNA was extracted by using the TransZol Up RNA extraction kit (Transgen Biotech, Beijing, China). The purities and concentrations of RNA were measured using a UV spectrophotometer (Biochrom, UK) with the absorbance 260/280 nm. PrimeScript^TM^ RT reagent kit with gDNA eraser (Takara, Dalian, China) was used to remove genomic DNA and reverse transcribe 1 *μ*g total RNA to cDNA. The target genes expressions were observed intuitively by the semiquantitative RT-PCR method. Agarose gel stained by GelStain (1%, Transgen Biotech, Beijing, China) was used to analyze the PCR products. In addition, quantitative PCR was performed in 20 *μ*L reaction volumes by qTOWER3G (Analytik Jena, Germany) to further quantify the expression levels of mRNA. The cycling procedure was 95°C for 30 s, followed by 40 cycles of 95°C for 5 s and 60°C for 30 s. The expressions of relative genes were analyzed according to the 2^−ΔΔCt^ method. The primer sequences are given in Table 1, and GAPDH was considered as an internal reference.

### 2.9. Western Blot Assay

RAW264.7 cells (1 × 10^6^ cells/dish) were cultured in 60 mm dishes for 20 h, CE was added in each dish (except for the control group) for 2 h of pretreatment, and 1 *μ*g/mL LPS was then added for incubation with indicated periods. RIPA lysis and PBS were used to prepare the protein lysates. The proteins were quantified using BCA kits, and 35 *μ*g proteins were further subjected to SDS-PAGE gels electrophoresis and transferred onto PVDF membranes. Then, they were blocked using 5% skim milk at room temperature for 1 h. The Super ECL Plus (US Everbright, Inc., Suzhou, China) was used to detect the protein bands by the ChemiScope 3300 system (Clinx, Shanghai, China) after the incubation of primary antibodies for 12–18 h at 4°C and the incubation of secondary antibodies at 24°C for 50 min.

### 2.10. Immunofluorescence Assay

The immunofluorescence assay for activity determination of NF-*κ*B p65 in this study was carried out by using the instructions of the NF-*κ*B activation-nuclear transfer kit (Beyotime, Shanghai, China). In short, RAW264.7 cells were inoculated into 60 mm dishes with 1 × 10^6^ cells/dish overnight. Then, RAW264.7 cells were cleaned and fixed with fixing solution for 15 min after the treatment of CE and LPS, followed by blocking with blocking solution for 1 h. The NF-*κ*B p65 primary antibody was added for 16 h incubation at 4°C, and the Cy3-conjugated secondary antibody was incubated for 50 min at 24°C. In the end, the cells were observed using the Eclipse Ts2 microscope (Nikon, Japan) after staining by DAPI.

### 2.11. Statistical Analysis

The data were processed by SPSS 25.0 (SPSS Inc., Chicago, IL, USA) and shown as mean ± SD values. The significance test was determined by one-way ANOVA followed by Tukey's multiple comparison tests, and *p*values <0.05 were considered as a significant difference.

## 3. Results

### 3.1. Effect of CE and Fr. 30% on Cell Viability

**Figure 1:**
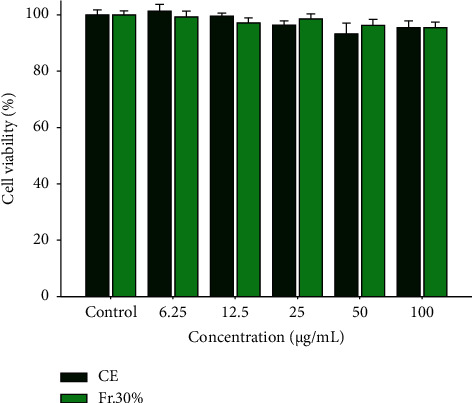
Cytotoxicity of crude extract (CE) and 30% fraction (Fr. 30%) with various concentrations (0–100 *μ*g/mL) on RAW264.7 macrophages (24 h of treatment). The cytotoxicity was determined by the MTT method. Data were presented as mean ± SD (*n* = 3).

The cytotoxicity of different concentrations of CE and Fr. 30% was assessed. The control group cells were regarded as 100% cell survival and other groups were compared with it. The results (Figure 1) suggested that both CE and Fr. 30% had high cell viability (over 90%) and no significant cytotoxicity with the concentration (6.25–100 *μ*g/mL). Therefore, the treated concentrations for subsequent studies were controlled under 100 *μ*g/mL.

### 3.2. Effect of CE and Fr. 30% on NO Production

**Figure 2:**
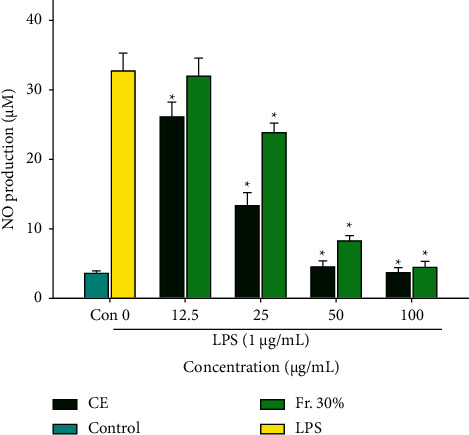
Effects of CE and Fr. 30% on NO release in RAW264.7 cells induced with LPS. The NO production was tested using the Griess reagent. Data were presented as mean ± SD (*n* = 3). ^*∗*^*P* < 0.05 vs. the LPS group.

The effects of CE and Fr. 30% with different concentrations on NO release in RAW264.7 cells were also studied in this work. As shown in Figure 2, the release of NO in the LPS group (32.74 ± 2.56 *μ*M) was significantly higher than the control group (3.58 ± 0.35 *μ*M) and indicated that LPS can successfully stimulate the inflammation of RAW264.7 cells. CE and Fr. 30% (25–100 *μ*g/mL) all prominently reduced NO production, and NO production decreased in a dose-dependent manner when the RAW264.7 cells treated with CE or Fr. 30%. There was no significant difference between 50 and 100 *μ*g/mL CE in inhibition of NO release. In addition, compared to Fr.30%, CE in various concentrations (12.5–100 *μ*g/mL) all showed higher inhibition of NO production. Thus, CE was chosen for further mechanism study.

### 3.3. Effect of CE on Cell Morphology of LPS-Induced RAW264.7 Cells

**Figure 3:**
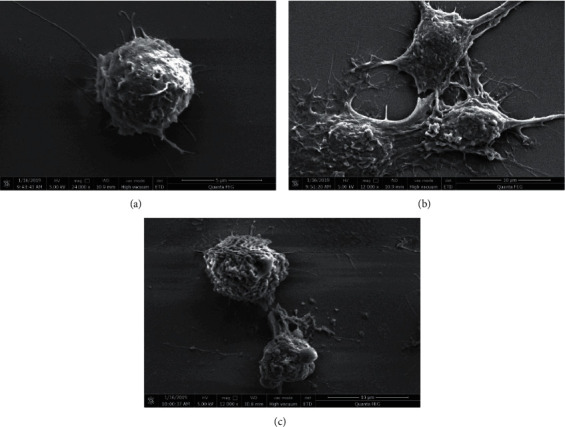
The morphology of RAW264.7 cells with LPS and CE treatment using SEM. (a) Control group cells. (b) LPS (1 *μ*g/mL) treatment cells. (c) LPS (1 *μ*g/mL) and CE (50 *μ*g/mL) treatment cells. The scale bar was 10 *μ*m and the original magnification was 12000 ×.

The changes in cell morphology of LPS-induced RAW264.7 cells were observed using SEM. It was evident that RAW264.7 macrophages in the control group (Figure 3(a)) exhibited normal morphology which were in circular shape with full forms and smooth surface. However, the morphology of RAW264.7 cells changed when LPS was added. Cells in the LPS-treated group (Figure 3(b)) had lots of dendritic pseudopods, and the morphology was spindle shaped and then became flattened; it suggested that RAW264.7 cells were activated. When 50 *μ*g/mL CE was given to the LPS-stimulated RAW264.7 cells, the number of the dendritic pseudopods was reduced and the cells morphology was transformed from flattened to smooth and full (Figure 3(c)). It indicated that the treatment of CE can attenuate the morphological changes when cells were induced by LPS.

### 3.4. Effect of CE on Proinflammatory Factors' Production

**Figure 4:**
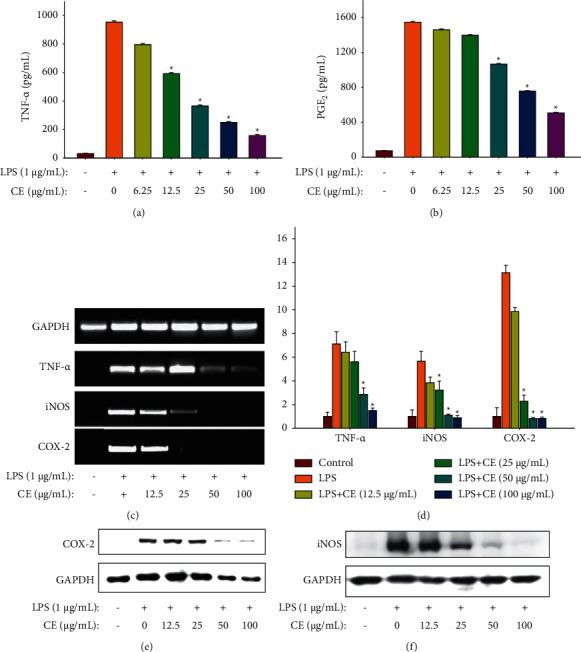
Effects of CE on proinflammatory factors production, related genes, and proteins expressions in LPS-induced RAW264.7 cells. Inflammatory factors production, related genes, and proteins expressions were determined by using ELISA kits, RT-PCR, and Western blot assay, respectively. (a) TNF-*α* production. (b) PGE_2_ production. (c) TNF-*α*, iNOS, and COX-2 mRNA expressions observed by semiquantitative RT-PCR. (d) TNF-*α*, iNOS, and COX-2 mRNA expressions determined by quantitative RT-PCR. (e) COX-2 protein expressions. (f) iNOS protein expressions. Data were normalized to GAPDH and presented as mean ± SD (*n* = 3). ^*∗*^*P* < 0.05 vs. the LPS group.

The concentrations of two proinflammatory factors including TNF-*α* and PGE2 were measured by ELASA kits. From Figures 4(a) and 4(b), it is clear that LPS treatment prominently increased the concentrations of PGE2 and TNF-*α*, whereas the concentrations gradually decreased when CE was added. With the increase in CE concentration, the secretion of TNF-*α* and PGE2 reduced significantly and exhibited a dose-effect relationship; it suggested that CE exerts anti-inflammatory activity according inhibition of inflammatory factors.

### 3.5. Effect of CE on Proinflammatory Factors' mRNA Expressions

Semiquantitative RT-PCR was applied to observe the mRNA expression intuitively and quantitative RT-PCR was used to measure the mRNA expression levels quantitatively. As shown in Figure 4(c), COX-2, iNOS, and TNF-*α* mRNA expression levels in the control group were extremely low, while the presence of LPS significantly increased these expression levels. TNF-*α* mRNA expression was strongly reduced with 50 and 100 *μ*g/mL CE. In addition, 25, 50, and 100 *μ*g/mL CE treatment all markedly inhibited COX-2 and iNOS mRNA expressions. Moreover, the results of quantitative RT-PCR (Figure 4(d)) were consistent with the semiquantitative RT-PCR (Figure 4(c)) and show the presence of CE-suppressed inflammation via downregulation of TNF-*α*, COX-2, and iNOS mRNA expression levels.

### 3.6. Effect of CE on COX-2 and iNOS Proteins' Expressions

The effect of CE in different concentrations on COX-2 and iNOS protein expressions in RAW264.7 cells was studied using Western blot. COX-2 and iNOS expressions were prominently upregulated after the treatment of LPS, whereas 50 and 100 *μ*g/mL CE all strongly downregulated their expressions (Figures 4(e) and 4(f)). Furthermore, CE at 100 *μ*g/mL nearly entirely suppressed iNOS protein expression, and the expression was almost downregulated to the basal level, which compared with the control group (Figure 4(f)). The results indicated that CE inhibited COX-2 and iNOS protein expressions to alleviate inflammatory responses.

### 3.7. Effect of CE on Regulation of Transcription Factors

**Figure 5:**
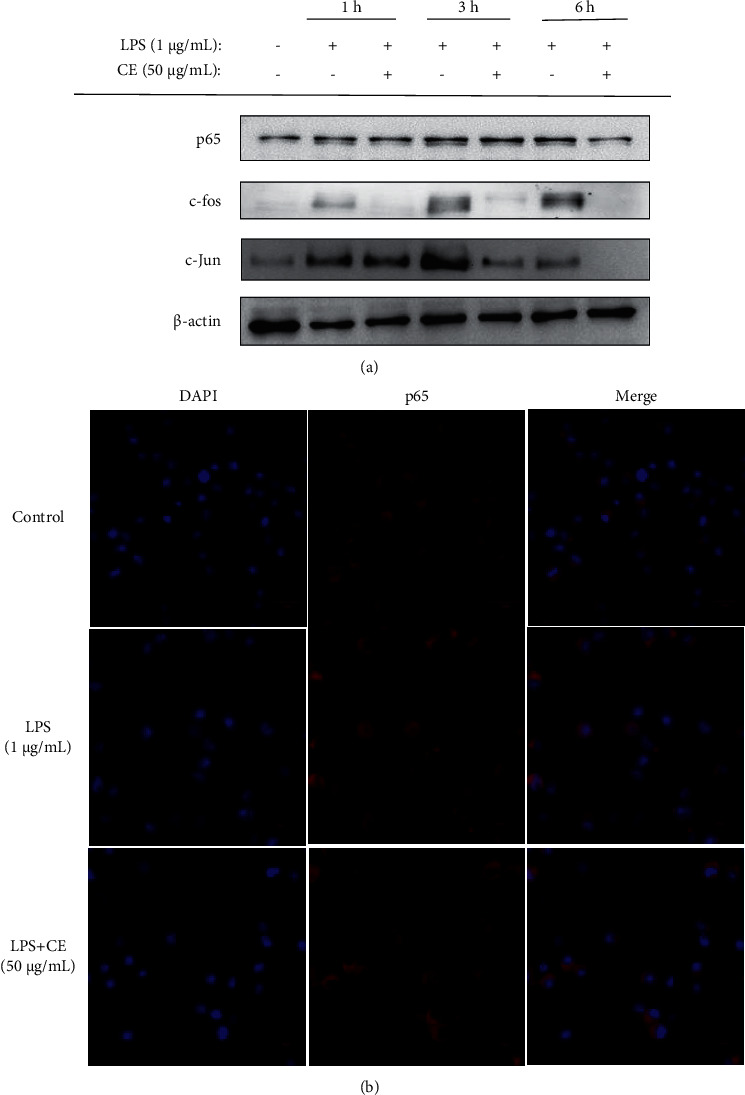
Effects of CE on nuclear translocation of transcription factors in LPS-induced RAW264.7 cells. (a) The expressions of p65, c-Fos, and c-Jun determined using Western blot. (b) The effects of 50 *μ*g/mL CE on translocation of NF-*κ*B p65 from the cytoplasm to nucleus using a confocal microscope.

The nuclear translocation of AP-1 and NF-*κ*B in LPS-induced RAW264.7 macrophages was examined in this work. AP-1 is a crucial transcriptional activator primarily composed of c-Jun and c-Fos [21]. As shown in Figure 5(a), the expression of c-Fos, c-Jun, and p65 was significantly upregulated with stimulation of LPS. However, 50 *μ*g/mL CE prominently downregulated c-Fos expression at 1 h, 3 h, and 6 h. Besides, 50 *μ*g/mL CE also markedly suppressed c-Jun expression at 3 h and 6 h. p65 is a key subunit of NF-*κ*B and its nuclear translocation was strongly inhibited after 6 h treatment of CE. In addition, a confocal microscope was further used to observe the effect of CE with concentration of 50 *μ*g/mL on p65 nuclear translocation. As shown in Figure 5(b), p65 in the nucleus was dramatically increased in the LPS group, activating NF-*κ*B. With the treatment of 50 *μ*g/mL CE, p65 entry from the cytoplasm into the nucleus was markedly inhibited. Collectively, CE alleviated inflammation by suppressing AP-1 and NF-*κ*B activation.

### 3.8. Effect of CE on Regulation of PI3K/Akt and MAPK Signaling Pathways

**Figure 6:**
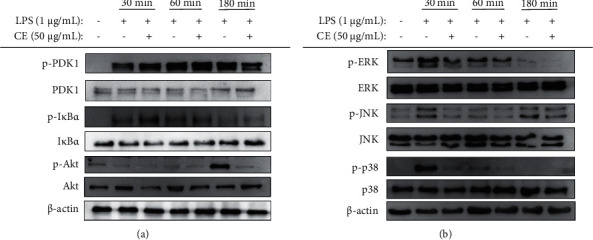
Effects of CE on PI3K/Akt and MAPK signaling pathways in LPS-induced RAW264.7 cells. RAW264.7 cells were pretreated with CE (50 *μ*g/mL) for 2 h and then treated with LPS (1 *μ*g/mL) for the indicated times. (a) The phosphorylation levels of PDK1, I*κ*B*α*, and Akt. (b) The phosphorylation levels of ERK, JNK, and p38.

Generally, NF-*κ*B expression alleviation is related to the PI3K/Akt and MAPK signaling pathway [5]. Hence, to verify whether the MAPK and PI3K/Akt signaling pathways were involved, RAW264.7 cells were treated with LPS and CE for different time points and the expression levels of p-PDK1, p-Akt, p-IkB*α*, p-p38, p-ERK, and p-JNK were measured. The expression levels of p-PDK1 and p-Akt all peaked at 180 min, while the treatment of 50 *μ*g/mL CE prominently reduced their phosphorylation (Figure 6(a)). Moreover, CE also downregulated the expression of p-IkB*α* at 180 min (Figure 6(a)). The phosphorylation levels of ERK, JNK, and p38 protein were markedly promoted, and all peaked at 30 min after the LPS was added, while 50 *μ*g/mL CE produced an obvious inhibition in their phosphorylation level at the same time (Figure 6(b)). Taken together, it suggested that CE attenuated inflammation via PI3K/Akt and MAPK signaling pathways downregulation.

## 4. Discussion

Inflammation, a complex pathophysiological response triggered by foreign microbial pathogens, is often accompanied with various diseases. LPS is the main component of the Gram-negative bacteria cell wall, which belongs to these foreign pathogens. It can potently activate RAW264.7 cells and trigger inflammation [22, 23]. Therefore, the LPS-induced RAW264.7 macrophages is a classical vitro cell model for the assessment and selection of some medicines in anti-inflammatory activity. GPL has been used in fever and inflammation treatment for a long time in history. In recent years, modern medical research has also reported its anti-inflammatory activity [8], but the research on its specific mechanism is still blank. This work will reveal its anti-inflammatory mechanism and provide data support for the utilization of GPL.

NO is a key inflammatory mediator closely associated with the inflammatory response. Low level of NO (<300 *μ*M) exerts anti-inflammatory effects, while excessive NO release can intensify the process of inflammation [7]. Therefore, the inhibition of NO release becomes a crucial indicator, reflecting the anti-inflammatory activity. GPL contains multiple active chemical components including rutin, astragalin, quercetin, and chlorogenic acid [17]. Among them, chlorogenic acid is the major active compound in GPL showing strong anti-inflammatory activity. Chlorogenic acid can prominently reduce the release of NO, TNF-*α*, and IL-1*β* by downregulating the expression of iNOS, Ninj1, and COX-2 and suppressing the nuclear translocation of NF-*κ*B [12]. In this work, the macroporous resin 306 was used to purify CE, and 30% ethanol was eluted in order to get a fraction with higher chlorogenic acid content (Fr. 30%). The contents of chlorogenic acid in CE and Fr. 30% were quantified via the external standard method using HPLC. The results showed that chlorogenic acid content in Fr. 30% was 43.7%, obviously higher than that in CE (10.5%). From this, we speculated that Fr. 30% was more effective in anti-inflammation owing to its high content of chlorogenic acid. However, intriguingly, the result of NO production suggested that Fr. 30% with various concentrations (12.5–100 *μ*g/mL) all showed lower inhibition of NO release, compared with CE (the concentration of CE and Fr. 30% from 12.5 to 100 *μ*g/mL was no cytotoxic) (Figures 1 and 2). The finding was quite contrary to our speculation. It also reminded us of the significance of crude extract and sparked our enormous interest in further investigations of whole crude extract. We considered that crude extract is rich in various active compounds, and the synergistic effect of these components may possibly contribute to the potent anti-inflammatory activity.

Besides NO, RAW264.7 cells can also increase the secretion of other proinflammatory factors, such as PGE2, IL-6, IL-1*β*, and TNF-*α* when stimulated with LPS. The secretion of these inflammatory factors derives from the related genes transcription and proteins translation, which are activated by a complex signaling cascade [24]. PGE2 and NO, as the pivotal regulatory factors in inflammation, majorly derives from arachidonic acid and L-arginine of COX-2 and iNOS proteins, respectively [25]. TNF-*α* can stimulate the secretion of other cytokines including IL-6 and IL-1*β* by the regulation of cytokine cascade, which in turn enhance the leukocytes recruitment to inflammation sites [26]. In this work, we discovered that 50 *μ*g/mL CE could prominently suppress the release of inflammatory factors including NO, PGE2, and TNF-*α* via inhibiting the expression of related genes and proteins, as confirmed by the result of RT-qPCR and Western blotting (Figure 4). Our results were largely in line with the study by Ning et al. [27], who pointed out that 250 *μ*g/mL *G. procumbens* extract was capable of reducing NO production by obviously downregulating iNOS protein expression. In addition, Huang et al. also reported that essential oils of *G. procumbens* markedly inhibited COX-2 overexpression to alleviate the inflammation and deswelling in mice [16]. The transcription of these inflammatory genes is regulated by NF-*κ*B and AP-1, which are important transcription factors [26]. Specifically, the activation of AP-1 and NF-*κ*B can promote the release of these inflammatory cytokines, which in turn makes a positive feedback loop to increase the AP-1 and NF-*κ*B [28]. Under normal conditions, I*κ*B*α* binds to NF-*κ*B composed of p50 and p65. When stimulated with LPS, I*κ*B*α* is activated and phosphorylated, followed by ubiquitination and degradation; then, NF-*κ*B is released, and the subunit p65 quickly enters to the nucleus to initiate or enhance the target gene transcription [29–31]. In our study, CE significantly reduced the phosphorylation of I*κ*B*α* (Figure 6(a)) and inhibited the nuclear translocation of AP-1 and NF-*κ*B (Figure 5). The activation of AP-1 and NF-*κ*B is regulated by intricate signaling pathways comprising MAPK and PI3K/Akt. The MAPK family contains three major signaling cascades, JNK, ERK, and p38 MAPK, relating to inflammatory reaction and cell growth, differentiation, and apoptosis [32]. Based on the discovery that CE suppressed the activation of NF-*κ*B and AP-1, the expressions of p-JNK, p-p38, and p-ERK were determined. The results indicated that CE downregulated MAPK signaling pathways when cells were stimulated. Furthermore, the phosphorylation levels of Akt and upstream PDK1 were also reduced, demonstrating that another signaling pathway PI3K/Akt was also involved in the inflammation attenuation.

## 5. Conclusion

In this work, we compared CE and Fr. 30% in anti-inflammatory effects and first elaborated on the latent mechanisms. The results displayed that CE exhibited a stronger anti-inflammatory effect, and this effect may derive to a greater extent from the synergistic effect of active components, rather than the content of a certain active compound. Moreover, CE markedly inhibited the secretion of proinflammatory factors via the downregulation of the MAPK and PI3K/Akt signaling pathways. Taken together, the extract of *G. procumbens* could be used for inflammation-related disorders alleviation, and these findings could provide the basis for the new functional food development.

## Figures and Tables

**Table 1:** Primers list.

Gene	Upstream primer sequence	Downstream primer sequence
TNF-*α*	5′-TGTCCCTTTCACTCACTGGC-3′	5′- CATCTTTTGGCGGAGTGCCT-3′
COX-2	5′- AGAAGGAAATGGCTGCAGAA-3′	5′- GCTCGGCTTCCAGTATTGAG-3′
iNOS	5′- CCCTTCCGAAGTTTCTGGCAGCAGC-3′	5′- GGCTGTCAGAGCCTCGTGGCTTTG-3′
GAPDH	5′- CACTCACGGCAAATTCAACGGCA-3′	5′- GACTCCACGACATACTCAGCAC-3′

## Data Availability

The data used to support the findings of this study are available from the corresponding author upon request.
